# P-94. Epidemiology, Clinical Characteristics, and Outcomes of Patients with Nontuberculous Mycobacterial Bone and Joint Infections at the University of Rochester Medical Center, New York from 2019-2023

**DOI:** 10.1093/ofid/ofaf695.323

**Published:** 2026-01-11

**Authors:** Patrick Passarelli, Sonal Munsiff, Alok Gupta, Michael Croix, Edward D Chan, Lauren Tapper, Dwight Hardy

**Affiliations:** Dartmouth, Lebanon, NH; University of Rochester, Rochester, NY; University of Rochester Medical Center, Rochester, New York; University of Rochester, Rochester, NY; National Jewish Health, Denver, Colorado; National Jewish Health, Denver, Colorado; University of Rochester Medical Center, Rochester, New York

## Abstract

**Background:**

Nontuberculous mycobacteria (NTM) bone and joint infections (BJI) are rare. They occur in immunocompetent and immunocompromised hosts often after direct inoculation via penetrating and non-penetrating trauma, surgery, or injections. Clinical cure often requires not only multi-drug regimens but also debridement or more extensive surgery. With little published data to guide management, we reviewed patients with NTM BJI over five years at our institution to better understand future diagnosis and treatment options.

**Figure:**
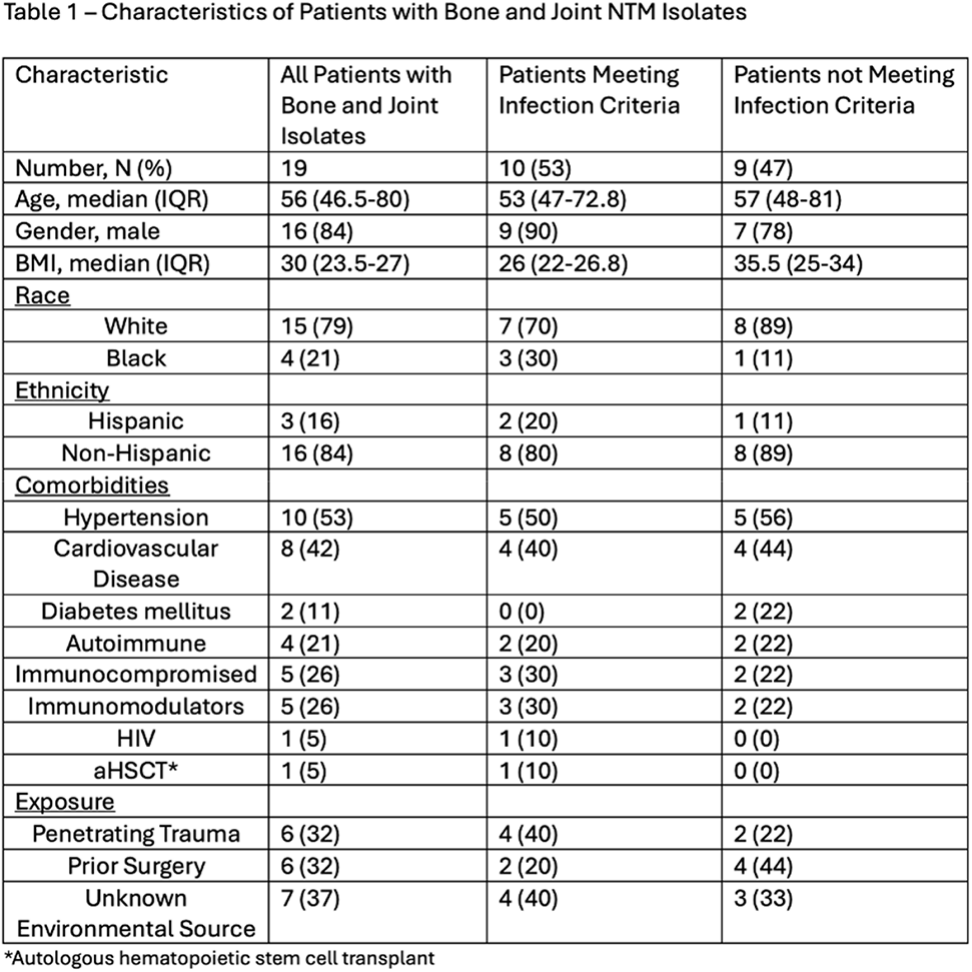

**Figure:**
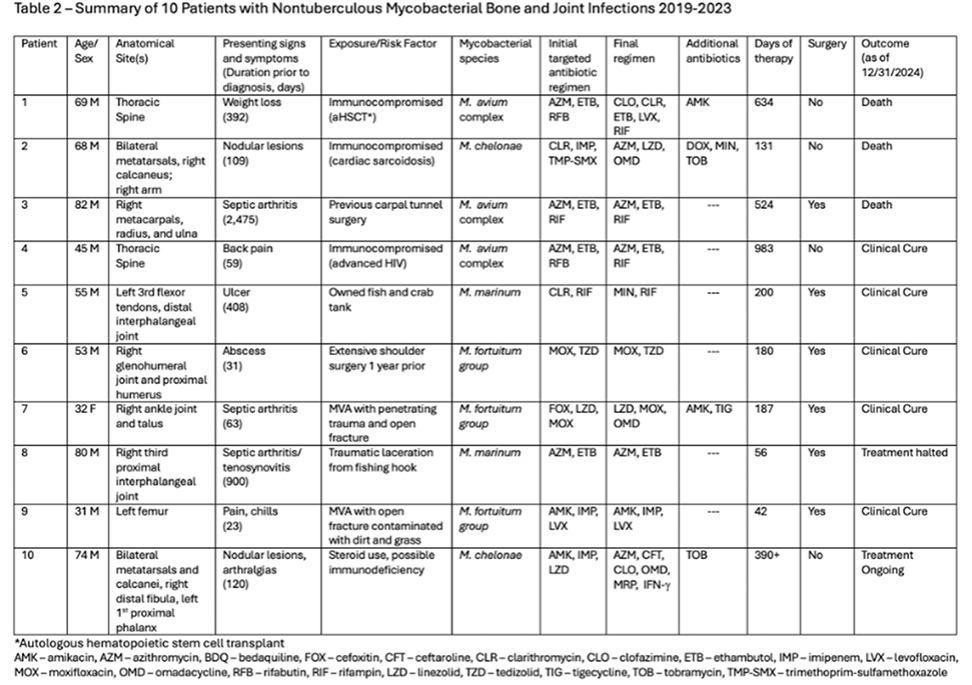

**Methods:**

Records of patients with NTM recovered from bone and joint cultures from 1/1/2019 thru 12/31/2023 were reviewed for clinical data from six months prior to report of NTM from index culture through 12/31/2024, or date of death if earlier. Descriptive statistics were calculated (Excel, v16.96.1).

**Figure:**
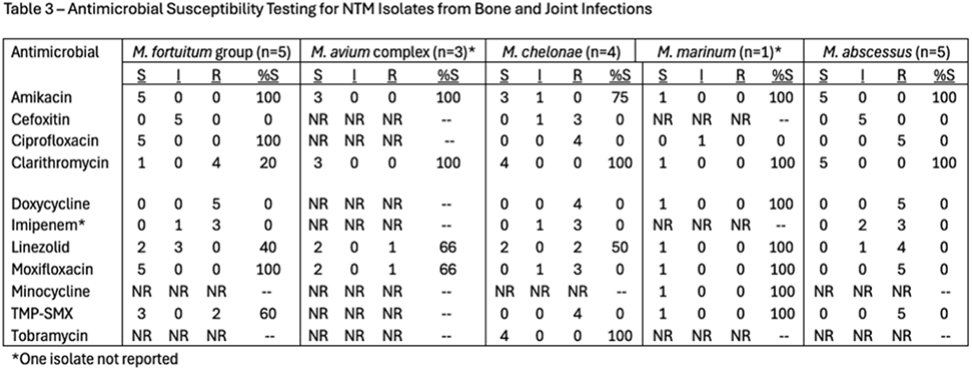

**Results:**

We identified 19 patients. Isolates from nine patients were deemed by treating clinician to be non-infection; 10 (53%) had infections. Nine (90%) were male; median age was 53 (range 31-92); 3 (30%) were immunosuppressed (Table 1).

Isolates included *M. fortuitum*, *M. avium*, *M. chelonae*, and *M. marinum*. Macrolide resistance was common for *M. fortuitum* isolates but not found in any other isolates (Table 3). Median time from symptom onset to diagnosis was 115 days (range 23 – 2,457). Median number of drugs used was 3 (range 2-9); median treatment duration was 194 days (range 42-983). Toxicity requiring change in therapy occurred in 30%. Management involved surgery in 60%. Death during therapy occurred in 30% (none attributed to NTM), one patient discontinued therapy after partial remission, one remains on treatment, and 50% had clinical cure. All patients < 60 years old were cured (median treatment 187 days); none >60 years had clinical cure; 3 (60%) died.

**Conclusion:**

Two distinct phenotypes of NTM BJI emerged: (i) younger patients with trauma-related exposure with shorter, less complicated treatment courses and high rate of clinical cure and (ii) older and/or immunosuppressed patients with presumed environmental exposure, longer treatment courses, higher rates of drug toxicity and death, and lower cure rates. Delay in diagnosis was common, especially in the latter group. Surgery was an important component of management in the majority of cases.

**Disclosures:**

Michael Croix, MD, DynaMed: Advisor/Consultant

